# The evolutionary origin of near-death experiences: a systematic investigation

**DOI:** 10.1093/braincomms/fcab132

**Published:** 2021-06-22

**Authors:** Costanza Peinkhofer, Charlotte Martial, Helena Cassol, Steven Laureys, Daniel Kondziella

**Affiliations:** Department of Neurology, Rigshospitalet, Copenhagen University Hospital, Copenhagen 2100, Denmark; Department of Psychiatry, Frederiksberg Hospital, Copenhagen University Hospital, Copenhagen 2000, Denmark; Coma Science Group, GIGA-Consciousness, University of Liège, Liège 4000, Belgium; Centre du Cerveau^2^, University Hospital of Liège, Liège 4000, Belgium; Coma Science Group, GIGA-Consciousness, University of Liège, Liège 4000, Belgium; Coma Science Group, GIGA-Consciousness, University of Liège, Liège 4000, Belgium; Centre du Cerveau^2^, University Hospital of Liège, Liège 4000, Belgium; Department of Neurology, Rigshospitalet, Copenhagen University Hospital, Copenhagen 2100, Denmark; Department of Clinical Medicine, University of Copenhagen, Copenhagen 2100, Denmark

**Keywords:** death, evolution, near-death experience, survival, tonic immobility

## Abstract

Near-death experiences are known from all parts of the world, various times and numerous cultural backgrounds. This universality suggests that near-death experiences may have a biological origin and purpose. Adhering to a preregistered protocol, we investigate the hypothesis that thanatosis, aka death-feigning, a last-resort defense mechanism in animals, is the evolutionary origin of near-death experiences. We first show that thanatosis is a highly preserved survival strategy occurring at all major nodes in a cladogram ranging from insects to humans. We then show that humans under attack by animal, human and ‘modern’ predators can experience both thanatosis and near-death experiences, and we further show that the phenomenology and the effects of the two overlap. In summary, we build a line of evidence suggesting that thanatosis is the evolutionary foundation of near-death experiences and that their shared biological purpose is the benefit of survival. We propose that the acquisition of language enabled humans to transform these events from relatively stereotyped death-feigning under predatory attacks into the rich perceptions that form near-death experiences and extend to non-predatory situations.

## Teaser

Thanatosis, also known as death-feigning or tonic immobility, is a well-described and phylogenetically highly preserved survival strategy in the animal kingdom. Peinkhofer et al. investigate the hypothesis that thanatosis is the evolutionary origin of near-death experiences in humans.

## Graphical Abstract

**Graphical Abstract:**
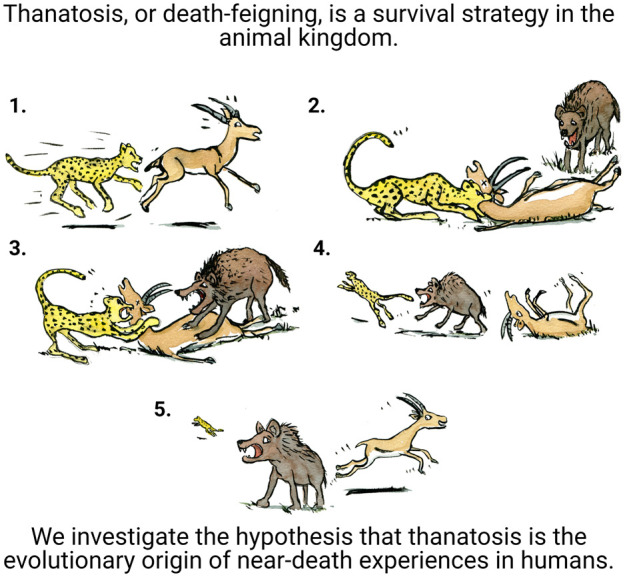

## Introduction

Near-death experiences (NDEs) are unique conscious, self-related emotional, spiritual and mystical unexplained experiences occurring in life-threatening situations or situations that may feel life-threatening, including cardiac arrests, traffic accidents, physical assaults and drug abuse.^1^ Typical elements of NDEs include distortion of time perception, increased speed of thoughts, life reviews, out-of-body experiences, feeling one with the universe, feeling peace and acceptance, sometimes even joy, and visual and auditory hallucinations, including seeing bright lights, being in a tunnel and meeting spirits.^1^

NDEs are not a rare phenomenon, occurring in around 10–23% of cardiac arrest survivors,^2–4^ in 3% of traumatic brain injury survivors,^5^ and in 4–8% of the general population (all causes combined).^6–8^ Although proposed NDE candidate mechanisms include cerebral N-methyl-d-aspartate receptor (NMDAR) hypofunction,^9^^,^^10^ intrusion of rapid eye movement (REM) sleep into wakefulness^11^^,^^12^ and migraine aura,^13^ the evolutionary origins of NDEs remain unknown.^1^ Given that NDEs have been recognized in various human civilizations for many centuries and from all inhabited continents, the question arises if NDEs may have a specific biological benefit. If this would be the case, then comparative biology might allow insights into the origins of NDEs.^14–16^

**Figure 1:**
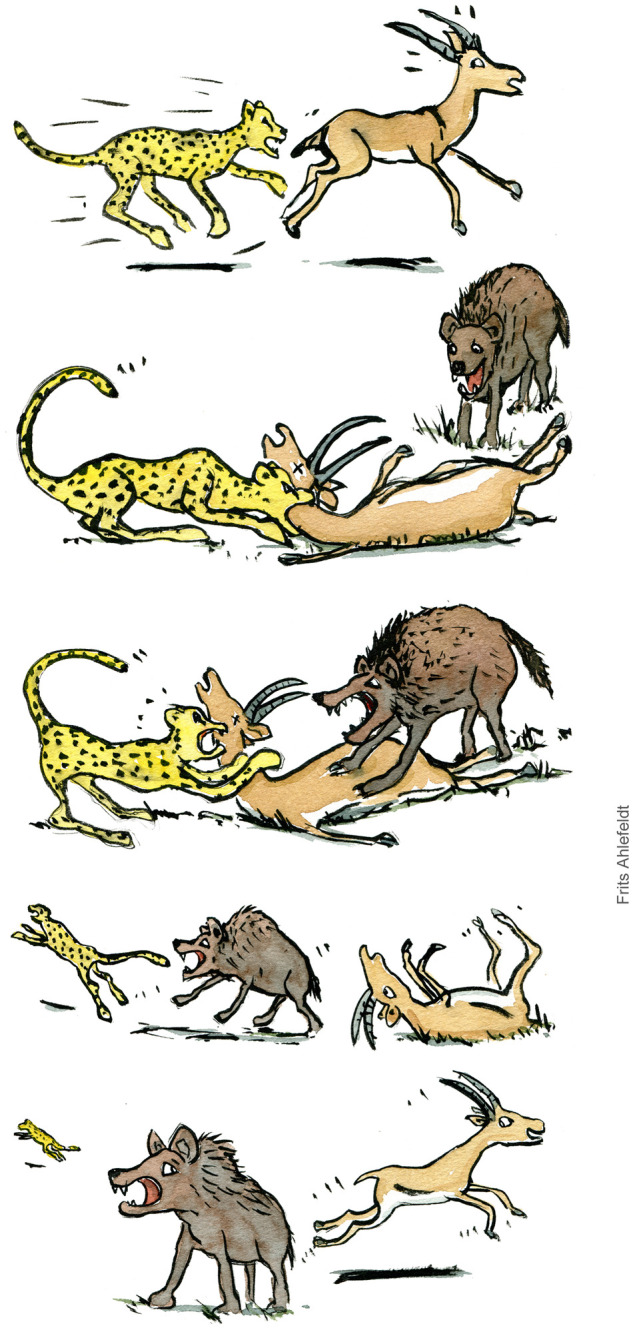
**Thanatosis increases the chances of survival.** Artist's impression of a video^111^ from the African savannah featuring a cheetah, a hyaena and an impala, illustrating the survival advantage of tonic immobility. The cheetah brings down an impala that lies apparently dead on the ground. A hyaena comes and takes over the prey. The hyaena examines the impala and bites it a few times (not shown), while the cheetah watches from a distance. Confident that the impala is dead, the hyaena chases the cheetah away, while the impala uses its chance to escape. Similar videos exist showing two impalas surviving attacks by a leopard and a hyena^112^^,^^113^ and a wild dog who escapes a lion.^114^ The artwork was created for the present article and published with permission by the artist, Frits Ahlefeldt, Copenhagen, Denmark.

When attacked by a predator, as a last resort, animals can feign death to improve their chances of survival (Fig. 1), one example being the opossum.^17^ This phenomenon is termed thanatosis, also known as death-feigning or tonic immobility.^18^ Thanatosis occurs in a large variety of taxa, including insects,^19^^,^^20^ reptiles^21^ and mammals.^22–24^ In humans, it has been described as a defense mechanism happening during sexual assault.^25^^,^^26^ Thus, thanatosis is an anti-predator strategy that is part of an innate defense cascade,^27^^,^^28^ which is activated when fight or flight are no longer possible.^14^^,^^29^^,^^30^ It involves sudden onset of immobility, with or without loss of tonic muscular activity, and unresponsiveness to external stimuli but preserved awareness.^30^ Of note, this is akin to some forms of REM sleep intrusion into wakefulness in humans, e.g. lucid dreaming and cataplexy, that can occur in NDEs.^12^

We hypothesized that NDEs originate from thanatosis and that thanatosis is phylogenetically preserved throughout the animal kingdom. Here, our aim was to conduct a systematic evaluation of the evidence to establish a line of argumentation that NDEs and thanatosis are heritable behavioural traits evolving under natural selection and serving the biological purpose of survival.

## Materials and methods

To investigate the association between NDEs and thanatosis, including phylogenetic aspects, we put together five work packages (WP 1–5). We registered the study protocol on 9 October 2020 with the Open Science Framework (https://osf.io/e8g7h), prior to data collection.

**WP 1:** The *objective* was to document the existence of thanatosis in animals at all major cladogram nodes. To this end, we first identified a suitable cladogram from insects to the great apes and humans, based on the National Center for Biotechnology Information (NCBI) taxonomy and created using freely available, non-commercial software (phylot.biobyte.de). We then performed a systematic literature search to identify at least 1–2 pertinent studies reporting on thanatosis or tonic immobility in the animal kingdom, for each branch of our cladogram. Briefly, we evaluated all cross-sectional or longitudinal, retrospective or prospective, observational clinical and research studies and reviews on thanatosis or tonic immobility in animals and humans. We searched MEDLINE, Scopus and Google Scholar for relevant English, French, German and Italian literature until 31 October 2020. The literature search was supervised by the library service of the University of Copenhagen. We used the search terms ‘thanatosis’, ‘thanatomimesis’, ‘death-feigning’, ‘tonic immobility’ and ‘apparent death’. References of relevant articles were manually searched to identify additional articles, and papers were cross-referenced using the ‘cited by’ function in PubMed. Search strategies (including MeSH headings) are available on request. Titles were reviewed first, followed by abstracts when titles suggested studies were relevant. Eligible studies were identified based on their full text. We selected 1–2 studies for each cladogram node, emphasizing reports showing a survival benefit with thanatosis. Furthermore, we discussed studies on thanatosis in monkeys with two behavioural ecologists (see Acknowledgements section).

**WP 2:** We searched the Liège Coma Science Group NDE database from the University of Liège in Belgium for NDEs related to physical assault, traffic accidents and similar events. The database was established in 2010. Participants are recruited through websites, social media, local news and publications of the Coma Science Group and are emailed questionnaires related to socio-demographic and NDE characteristics. The *objective* was to document the occurrence of NDE in humans under attack by human predators such as sexual offenders and ‘modern’ predators such as approaching cars in traffic accidents.

**WP 3:** We reached out to NDE communities via Facebook, Instagram and Twitter to inquire about NDEs related to encounters with big animals (e.g. sharks, tigers). The *objective* was to document the occurrence of thanatosis and NDEs in humans under attack by animal predators.

**WP 4:** We contacted suitable organizations that track encounters between humans and big animals, including sharks (Taronga Zoo in Sydney, and similar institutions in South Africa, Florida and California), African wildlife (e.g. Serengeti National Park) and tigers (e.g. the Nagarjunsagar-Srisailam Tiger Reserve in India), to inquire about possible NDEs in survivors of these encounters, and searched the Internet using Google and Google Scholar, for similar reports of NDEs happening during human encounters with big animals. The *objective* was to document the occurrence of thanatosis and NDEs in humans under attack by animal predators.

**WP 5:** We searched through testimonials of survivors of mass executions and similar atrocities during the Holocaust, the war in Ex-Yugoslavia, and the Rwanda genocide, and terrorist attacks within the past 10 years, for examples of thanatosis and/or NDEs that might have helped these people to survive the events. Furthermore, we interviewed a survivor of the Auschwitz concentration camp (see *Acknowledgements*); we contacted and searched dedicated websites from relevant institutions such as the *United States Holocaust Memorial Museum*; *Yad Vashem* and *Remembering Srebrenica*; and we searched for testimonials using Google and Google Scholar. The *objective* was to document the occurrence of thanatosis in humans leading to a survival benefit.

### Ethics

The Ethics Committee of the Capital Region of Denmark waives approval for online surveys and inquiries. NDE testimonies of the Coma Science Group database were collected with approval by the ethics committee of the University of Liège.

### Data availability

Raw data are available from the authors on request.

## Results

**Figure 2:**
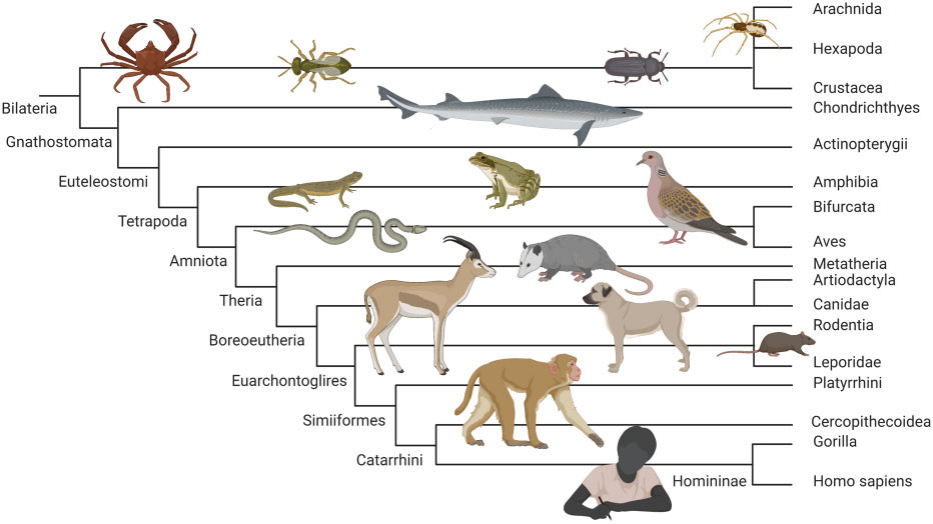
**Thanatosis is well preserved though evolution.** This figure depicts a cladogram, based on the National Center for Biotechnology Information (NCBI) taxonomy, ranging from insects and other arthropods to humans. Selected examples of animals for which there is evidence of thanatosis and a survival benefit are placed on each branch of the cladogram (from left upper to right lower corner): Northern kelp crab (*Pugettia producta*), Nasonia wasp (male), mealworm beetle (*Tenebrio molitor*), a spider (*Oedothorax retusus*), a gummy shark (*Mustelus antarcticus*), Eastern newt (*Notophthalmus viridescens*), a wood frog (*Rana sylvatica*), European turtle dove (*Streptopelia turtur*), a grass snake (*Natrix natrix*), Virginia opossum (*Didelphis virginiana*), Dorcas gazelle (*Gazella dorcas*), Kangal shepherd dog, a rat (*Rattus norvegicus*), Rhesus macaque (*Macaca mulatta*), and a human being (*Homo sapiens*). Derived characters at the nodes include the following: bilateral symmetry (Bilateria), invertebrate animals with exoskeleton, a segmented body, and paired jointed appendages (Arthropoda), jawed vertebrates (Gnathostomata), four-limbed animals (Tetrapoda), group of reptiles (Sauria), animals with amnios (Amniota), mammals giving birth without a shelled egg; including placental and marsupials (Theria), clades based on molecular analysis (Boreoeutheria and Euarchontoglires), Old World monkeys and apes (Catarrhini), gorilla, humans, chimpanzees and bonobos (Homininae). Figure created with biorender.com.

**WP 1:** We showed that thanatosis occurs at all major nodes in a NCBI taxonomy-based cladogram (ranging from insects, reptiles and birds to mammals, including humans), and is associated with a survival benefit (Fig. 2).

Our literature search yielded 16.266 titles. After screening and removal of duplicates, 32 articles were included. We found at least one article for all the branches of the cladogram. Two articles were selected for Arachnida,^31^^,^^32^ Hexapoda,^29^^,^^33^ Crustacea,^34^^,^^35^ Chondrichthyes,^36^^,^^37^ Actinopterygii,^38^^,^^39^ Amphibia,^40^^,^^41^ Bifurcata,^21^^,^^42^ Metatheria,^17^^,^^43^ Artiodactyla,^44^^,^^45^ Canidae,^46^^,^^47^ Rodentia,^23^^,^^48^ Leporidae,^49^^,^^50^ Cercopithecoidea^51^^,^^52^ and *Homo sapiens*.^25^^,^^26^ Three articles were selected for the Aves class.^53–55^ One article was found for the Platyrrhini^56^ and none for the genus *Gorilla*.

In three articles, death-feigning was observed in the field during a predator attack^45^^,^^46^ and during a cockfight.^55^ Eight articles reported thanatosis during a predatory attack in a research setting.^17^^,^^29^^,^^31^^,^^33^^,^^40^^,^^43^^,^^53^^,^^54^ In 17 papers, death-feigning and tonic immobility were evoked through manipulation or restraint of the animal by a study investigator.^21^^,^^23^^,^^36–42^^,^^44^^,^^47–52^^,^^56^ Finally, in four papers, death-feigning was recorded in reaction to different types of simulated threats, such as air puffs, grasping and touching with a stick.^32^^,^^34^^,^^35^^,^^40^ In the species *H.* *sapiens*, tonic immobility happened with traumatic events, including sexual assaults, war and torture.^25^^,^^26^

**Figure 3:**
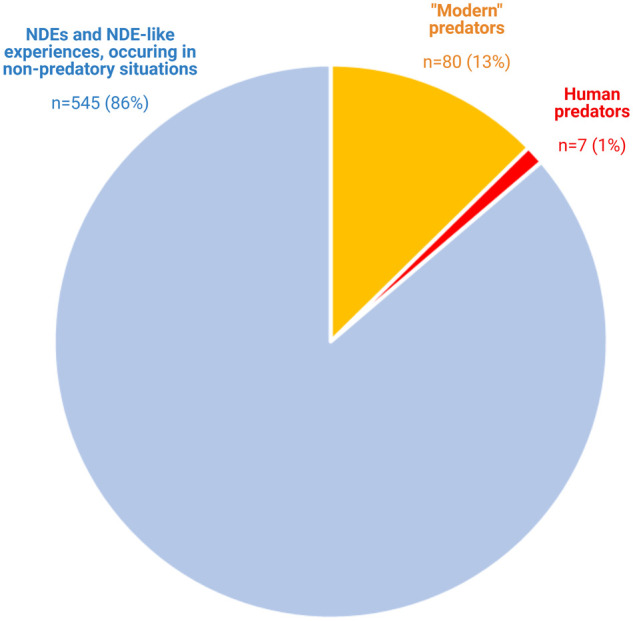
**Near-death experiences can occur with attacks from human and ‘modern’ predators.** Pie chart showing data from the NDE database of the Coma Science Group in Liège, Belgium. Depicted are the proportion of NDEs and NDE-like experiences related to predatory versus non-predatory causes (*n* total = 632). NDE-like refers to experiences made in situations without any obvious danger of death, e.g. syncope. Eighty-seven (14%) of 632 NDEs and NDE-like experiences occurred during encounters with human or ‘modern’ predators: In 7 cases (1%) these predators were other humans, including 3 cases of attempted murder, 1 case of sexual abuse and 3 armed robberies; and in 80 cases (13%), ‘modern’ predators were inanimate objects such as cars and other traffic vehicles.

**WP 2:** The Coma Science Group NDE database currently includes testimonies from 632 participants (342 females; mean age at NDE = 32 ± 17 years; mean age at interview = 57 ± 14 years; Greyson NDE total score = 15 ± 7). Participants are French, Dutch or English speakers and live in Europe or North America. Figure 3 contains the proportion of the different types of NDEs in this database. The present sample included 545 (86%) experiences from situations unrelated to predatory attacks, like cardiac arrest/anoxia (*n* = 111), anesthesia/surgery (*n* = 70), non-traumatic events such as septic shock (*n* = 189), and traumas including falls (*n* = 48), as well as NDE-like experiences (i.e. experiences without obvious threat to life) such as fainting (*n* = 127). By contrast, 87 (14%) NDEs occurred in situations involving human or ‘modern’ predators. Among these, 7 (1%) occurred with physical assault by a human predator (1 sexual abuse, 3 armed robberies and 3 attempted murders), and 80 (13%) occurred following encounters with ‘modern predators’ (e.g. vehicles involved in traffic accidents) (Fig. 3).

**WP 3–4:** Reaching out to NDE communities via social media and contacting organizations tracking human encounters with big animals, we were unable to identify volunteers with NDEs. We contacted big animals sanctuaries (Tigerheaven and Lions; Tigers and Bears), national parks (Serengeti National Park, Masai Mara National Reserve, Kruger National Park, Tarangire National Park, Madikwe Game Reserve, Satpura National Park, Madhya Pradesh Forest Department, and Mudumalai Tiger Reserve), zoos (ZooAmerica, Taronga Zoo) and research centres (Florida Program for Shark Research, and Taronga Conservation Society, Bandipur Tiger Project, Wildlife Investigation Lab). Most of the time, our inquiries remained unanswered despite repeated attempts, or we were told that our request could not be processed for unspecified reasons. However, a cursory Google-based search revealed reports of thanatosis (Box 1) and NDEs (Box 2) related to encounters with two lions, a great white shark, a tiger and a grizzly bear.

Box 1. Tonic immobility in encounters with big animals

**An attack by a grizzly bear** ^106^

“*Around 2 a.m. I had been very sound asleep, and I had this sense that something was badly wrong, and it was bringing me out of my sleep. I was just becoming aware, and the bear clamped down on my arm. The tent was gone at that point. Then the bear bit down and held me there for a while. […] Then I started yelling and the first thing I yelled was, “Oh no!” It was really an unbelievable moment for me. The most bizarre things go through your head. I knew I was in big trouble. The more I yelled, the more aggressive the bear got. […] I figured: I am definitely prey. At that point my gut told me not to fight. […] I knew my bear spray was behind me; I didn’t have a whole lot of options. So I tried to play dead and see what happened. […] When I decided that the only option was to play dead, I just went limp. Like a rag doll, didn’t move a muscle, didn’t move an eyelid. You can disassociate yourself from what’s going on. […] I was listening, and I could hear the people in the site next door make a run for the car. They got into the vehicle, slammed the door, and I heard the click click [of the lock before they drove off]. The bear dropped me sometime around then. Later on, when I thought about it, [the click] was what made the bear move off. I didn’t hear the bear leave. But when the bear dropped me I didn’t move for quite a while; I didn’t move for fear it might pounce on me.*”

**An attack by a lion** ^107^

David Livingston (1813–1873), famous 19th century Africa explorer. *“Starting, and looking half round, I saw the lion just in the act of springing on me. I was on a little height; he caught my shoulder as he sprang, and we both came to the ground below together. Growling horribly close to my ear, he shook me as a terrier dog does a rat. The shock produced a stupor similar to that which seems to be felt by a mouse after the first shake of the cat. It caused a sort of dreaminess, in which there was neither sense of pain nor feeling of terror, although quite conscious of all that was happening. It was like what patients partially under the influence of chloroform describe, who see all the operation, but feel not the knife. This singular condition was not the result of any mental process. The shake annihilated fear, and allowed no sense of horror in looking round at the beast. This peculiar state is probably produced in all animals killed by the carnivora; and if so, it is a merciful provision by our benevolent Creator for lessening the pain of death.”*

Box 2. Near-death experiences in encounters with big animals

**An attack by a shark** ^108^

***‘*** *When she was about twenty-four, Sherry survived a shark attack off the coast of South Padre Island, Texas. She was pulled under the surface of the water three times by her monstrous adversary. “I saw the horror in the face of the swimmers coming to rescue me before the huge ‘something’ grabbed me for the second time. I thought I would surely die. [..] When [the shark] pulled me under for the third time, I was shown a review of all the major scenes of my life. It is just incredible to think how you can see all your life in what is perhaps only two or three seconds of linear time.”’*

**An attack by a tiger** ^109^

Roy Horn (1944–2020), famous 20th century illusionist. ‘*On the operating table [during emergency surgery immediately after a tiger attack], Horn told Shriver he had a near-death experience. “I saw a bank of white light, and then I saw all my beloved animals,” Horn said. “For a moment I stepped out of my body*.”’

**An attack by a lion** ^110^

*‘After walking five steps into the cage, a lioness jumped up and attacked me. As I blacked out, the lioness took three bites to my head. Then I turned around and she bit me on the side of the head. Then finally she bit my chest, my right breast, then I lost all consciousness. While I was in this unconscious state, I went through the most amazing beautiful blissful experience. I saw things about me and my family. I saw my things from the future, like my 19-year-old brother with three baby girls pleading with me for help. I saw my entire life. I remember small bits of it now, but barely anything. I went to this amazing beautiful place: Some call it heaven; some call it God; some call it hallucinating. All I know is that this is where everyone truly belongs. It is where the soul goes. I wanted to stay but then something happened. I heard a voice, my voice, kept saying, ‘YOU’RE ONLY 15. GET UP, RUN!' Then the blissful place in which I was in, closed. It was like I was in a portal that looked like a black hole. It was a black hole with all the colours you can imagine and colours that the human eye does not recognize, and it closed.’*

**WP 5:** Searching dedicated websites and newspapers articles, contacting relevant institutions, and interviewing a former Auschwitz concentration camp prisoner, we found examples of (voluntary) death-feigning in survivors of the Holocaust (1941–45; e.g. the mass executions at Babi Yar in Kyiv, Ukraine, September 1941,^57^ and in the Budapest Ghetto, Hungary, January 1945),^58^ the Rwanda genocide (1994),^59^ and the Srebrenica massacre (1995),^60–62^ as well as the Utøya terrorist attack in Norway (2011)^63^^,^^64^ and the Orlando nightclub shooting in the USA (2016).^65^

## Discussion

We used a pre-registered, systematic and multilayered protocol to investigate the hypothesis that thanatosis, aka death feigning or tonic immobility, is the evolutionary origin of NDEs. First, we constructed a cladogram, ranging from insects to humans, based on NCBI taxonomy. We then systematically reviewed the scientific literature to show thanatosis exists in species from all branches of the cladogram, which suggests that it is a highly preserved phylogenetic trait. Furthermore, we showed thanatosis is associated with a survival advantage in animals as well as humans. Finally, we showed that humans under attack by big animals, other humans and ‘modern’ predators can both exhibit thanatosis and have NDEs, suggesting that these two conditions not only share important features but are related. We hypothesize that the greater sophistication of the human brain and the acquisition of language enabled humans to record and share their experiences in detail with others, thereby transforming these events from relatively uniform tonic immobility into the rich perceptions that form NDEs and extend to non-predatory situations.

### Thanatosis and the benefit of survival

Thanatosis is an anti-predator strategy and the terminal defense response when other options of fight or flight are futile.^29^ It is characterized by sudden immobility, with or without loss of tonic muscular activity, and unresponsiveness to external stimuli, while awareness is preserved.^30^ Awareness is necessary to be able to react when the chance to escape from imminent danger suddenly comes, against all odds (Fig. 1).

We found numerous examples within the animal kingdom that playing dead saves lives. Furthermore, we showed that thanatosis occurs in taxa at all important nodes in our cladogram, ranging from invertebrates^66–69^ to vertebrates,^21^^,^^41^ including mammals^22^^,^^23^ and humans^70^ (Fig. 2). This confirms thanatosis is a highly preserved biological phenomenon, and it suggests that thanatosis as a survival mechanism is probably phylogenetically as old as the fight-or-flight response.^71^

Being a heritable behavioural trait, thanatosis can evolve under natural selection for fitness of survival.^33^^,^^72^ This is true between and within species. For instance, Miyatake et al.^33^ artificially selected red flour beetles (*Tribolium castaneum*) for their ability to feign death. After ten generations, beetles selected for their death-feigning behaviour survived encounters with a predator, a female Adanson jumper spider (*Hasarius adansoni* Audouin), significantly more often than beetles with poorly developed tonic immobility.^33^ In a follow-up experiment, using a related species, *Tribolium freemani*, as prey and a predatory bug as predator, the authors found beetles selected for longer durations of death feigning had higher survival rates and longer latency to being preyed on when they were placed with predatory bugs than beetles selected for shorter durations of death feigning. Moreover, wild beetles from places where predators were abundant feigned death longer than wild beetles from predator-free populations. In sum, these experiments provide evidence that predators drive the evolution of death feigning.^72^

Despite these recent data, the notion of thanatosis offering a hereditable benefit for survival is not new. Already Charles Darwin commented on death feigning, conscious or unconscious; and the purpose of survival:

Animals feigning, as it is said, death—an unknown state to each living creature—seemed to me a remarkable instinct. […] I am inclined to think that in many instances it is a conscious simulation of death, adopted by the animals from the instinctive knowledge of the fact that certain birds and beasts of prey, except under pressure of extreme hunger, will not attack what is dead […] Now it will not be disputed that [this] is useful to each species, according to the kind of danger which it has to escape; therefore there is no more real difficulty in its acquirement, through natural selection, of this hereditary attitude than of any other.^73^

### The link between thanatosis and NDEs

Given the greater sophistication of the human brain including, notably, the evolution of language, it seems conceivable that in *H.* *sapiens* thanatosis would evolve from a relatively stereotypical behaviour into a more elaborate experience with rich details that can be reported and shared with others (even though less eloquent individuals may describe NDEs as ineffable), and which also may extend to situations other than predatory attacks, i.e. NDEs.

Although the association between thanatosis and NDEs remains difficult to prove beyond doubt, we showed that both thanatosis and NDEs occur in humans under attack by big animals and that the associated narratives are very similar (Box 1 and Box 2). As modern humans no longer have natural enemies, it should be no surprise that thanatosis and NDEs occur even more frequently in encounters with human and ‘modern’ predators. Such predators are sexual offenders, armed robbers, terrorists, prisoner guards and enemy soldiers, or inanimate objects, such as cars in traffic accidents. Thus, thanatosis as a self-defence mechanism has been well-described in victims of sexual assault,^70^ and in WP2 14% of the reported NDEs occurred in situations involving ‘modern’ or human predators (Fig. 3).

Hallucinations occurring in victims of predatory aggression are well-described from the Rwanda genocide, the Srebrenica massacre and the Holocaust (see for example^74^ for the Srebrenica massacre), but we were unable to identify classical NDEs in genocide survivors. Of note, however, we did find examples from each of these three events when (conscious) death feigning enabled individuals of Tutsi, Bosnian Muslim or Jewish heritage to survive mass murder against all odds. A similar survival strategy allowed people of Scandinavian descent to survive the Utøya terrorist attack in Norway,^64^ suggesting that death feigning—conscious or not—is a survival strategy irrespective of cultural and ethnic backgrounds.

We can conclude that, in analogy to the archaic ‘fight or flight’, also in humans there are evolutionary preserved cerebral mechanisms involving death-feigning for self-defence. It seems not to make a substantial biological difference if death is feigned as an involuntary or a (semi-)conscious act. What counts is that victims are lying still to increase their chances to survive the event. We suggest that some will do it while being fully aware; others will enter a state of dissociation which helps them to cope with the situation (Boxes 1 and 2). Even others may experience that fright and panic turn into peacefulness and sensory percepts that together constitute an NDE.

The question remains why NDEs occur in non-predatory situations such as with resuscitation during cardiac arrest. Several authors have speculated about a possible survival value of NDEs in these situations. We review their suggestions here before offering our own opinion.

Pfister^75^ and Noyes^76^ argued that pleasurable death fantasies in critical situations, including depersonalization and out-of-body experiences, protect the individual from being paralyzed by emotional shock.^75^^,^^76^ Similarly, Krishnan proposed that the elaborate cognition of NDEs maintains input to the brain, providing a homeostatic function, while sensory input has ceased due to progressive cerebral dysfunction.^77^ Greyson^78^ suggested that the peaceful affect and behavioural relaxation in NDEs may conserve energy reserves and prolong life in a situation where panic or agitation might rapidly deplete energy reserves.

In contrast to these authors, we suggest that the survival benefit of NDEs is limited to predatory situations and that NDEs in non-predatory situations may have no such purpose. Corroborating this idea, the human behavioural repertoire comprises a variety of behaviours which are phylogenetically highly preserved but whose benefits are restricted to certain situations. Examples include yawning and laughing when being tickled.

Evolutionary biologists and neuroscientists have suggested that mammals, including humans, evolved laughing in response to tickling to signal submission to an attacker or to foster parent-child interactions,^79^^,^^80^ and yawning can be useful in synchronizing the behaviour of a social group, for example, to get members of a heard to sleep at the same time.^81^ While the benefit of yawning for humans seems obvious during childhood (i.e. parents are triggered to put their baby to sleep), adults often suppress the urge to yawn given its negative social stigma (e.g. yawning during a conversation with the boss is unlikely to be beneficial). Similarly, most adults perceive the urge to laugh when tickled as a nuisance.

In the same vein, we think that the cerebral mechanisms behind NDEs have evolved from thanatosis because they offer a survival benefit during predatory attacks (after all, if an event is not survived, it has not been an NDE), but this pertains only to a minority of life-threatening situations. Since humans no longer have natural enemies, in most life-threatening situations (or situations that are perceived as such) NDEs are unlikely to have a specific biological purpose or their benefit might be less obvious.

### Biological mechanisms

Several neuronal candidate mechanisms have been proposed to contribute to NDEs, including cortical spreading depolarizations (CSDs),^13^ REM sleep intrusion into wakefulness^12^ and NMDAR hypofunction.^10^ In analogy to the above argumentation, some of these mechanisms may also apply to thanatosis.

CSDs are an attractive NDE candidate mechanism because a short-lasting variant of CSDs is considered the pathophysiological correlate of migraine aura,^82^ while terminal CSDs occur in humans at the end of life.^83^^,^^84^ Indeed, terminal CSDs almost invariably occur during the dying process of any creature with a brain, including humans,^83^^,^^84^ rats^85^ and insects.^86^ One of us therefore recently suggested that terminal CSDs are a phylogenetically preserved mechanism which must have occurred in the last common ancestor of humans and insects for over 500 million years ago.^14^ CSDs might therefore be conceivable as an underlying mechanism for both NDE and thanatosis. Indeed, migraine aura (which is caused by CSDs) was a predictor of NDE in a crowdsourcing study^13^ of unprimed lay people adjusted for age and sex (OR 2.33, *P* < 0.001). However, the low speed with which CSDs spread along the cortex, ∼3.2 mm/min,^87^ seems incompatible with the instantaneous shift from tonic immobility to full flight which in the end allows the impala from Fig. 1 to escape its predators.

In contrast to CSDs, REM sleep intrusion into wakefulness, which often includes cataplexy, happens abruptly, is instantaneously reversible^88^ and therefore could be an underlying mechanism for the rapid transition from flight to tonic immobility and back to flight again. Neurons of the periaqueductal grey and the adjacent deep mesencephalic reticular nucleus are essential for the control of sleep-wake state and components of the flip-flop circuit that maintains sleep bistability, which includes REM sleep.^89^^,^^90^ Importantly, a similar flip-flop mechanism involving the midbrain periaqueductal grey matter has been implicated in fight-and-flight responses.^91^ Active coping strategies, such as fight and flight are believed to be evoked by activation of either the dorsolateral or the lateral columns of the periaqueductal grey. In contrast, activation of the ventrolateral periaqueductal grey is thought to lead to passive coping like tonic immobility and decreased responsiveness to environmental stimuli.^91^^,^^92^

REM sleep intrusion is also an attractive NDE candidate mechanism because it is a natural phenomenon that occurs several times each night in everyone; it is associated with dissociative features including muscle atonia and hallucinations^93^; REM sleep intrusion into wakefulness is a feature of narcolepsy as well as healthy people^88^; and lucid dreaming and cataplexy which are features of REM sleep intrusion into wakefulness^93^ can occur in NDEs.^12^ Furthermore, REM sleep and REM sleep-like electrophysiological phenomena occur in a large variety of mammals^94^ and non-mammalian vertebrates, such as birds, lizards and fish.^95^ We can therefore conclude that also these mechanisms are phylogenetically well-preserved.

So far, two studies have investigated the association of NDE with REM sleep.^11^^,^^12^ In a case–control study, the prevalence of REM sleep intrusion was 60% in a sample of people with NDE and 24% in controls.^11^ A crowdsourcing study of >1000 unprimed laypeople from 35 countries confirmed an association between the two conditions: While age, sex, place of residence, employment status and perceived threat did not influence the prevalence of NDEs, people with REM intrusion were much more likely to report NDEs than those without (OR 2.85, *P* < 0.0001).^12^

Tonic immobility occurs in several conditions with altered consciousness, e.g. hypnosis, psychologically dissociative states and NMDAR hypofunction. The latter is induced by drugs, notably ketamine,^96^ or autoimmune mechanisms such as NMDAR encephalitis.^97^ NMDAR hypofunction is yet another attractive candidate mechanism that links tonic immobility in animals with NDEs in humans. To assess the neurochemical underpinnings of NDEs, Martial et al.^10^ searched 15 000 written consumer reports on 165 psychoactive substances and 625 NDE narratives semantic similarities, using a text mining approach. The substance most frequently associated with NDE-like reports was ketamine. Supporting the importance of NMDAR hypofunction in NDEs, ketamine is associated with dissociative properties^98^ which are, as stated, a well-established feature of NMDAR encephalitis^99^; and abuse of ketamine for recreational purposes can induce NDEs.^10^

Writing in *Nature*, Vesuna et al.^100^ investigated th*e* cellular and network mechanisms by which ketamine might induce its dissociative features in the brain. The authors recorded brain-wide neuronal activity in mice using wide-field calcium imaging and studied changes in brain rhythms in response to ketamine. This drug, but not others without dissociative properties such as propofol and LSD, produced robust 1–3 Hz oscillations of neuronal activity in layer 5 of the retrosplenial cortex. This is an essential brain area for various cognitive functions, including visuospatial navigation and episodic memory. When recording neuronal activity across multiple brain regions, the authors further found that ketamine caused a disconnect of the retrosplenial cortex in such a way that this area no longer communicated with others.^100^ Next, the authors investigated mice who had not received ketamine but whose layer-5 cells had been modified so that an artificial 2 Hz rhythm was produced. These mice showed the same dissociative behaviour as mice treated with ketamine, i.e. they did not rear away from threats or attempt to escape when suspended by their tails, although they still responded to painful stimuli. This confirmed these oscillations were indeed responsible for the observed dissociated state. Finally, to investigate if identical oscillations can induce dissociation in humans, Vesuna et al. recorded electrical activity from several brain regions in a patient with epilepsy, who experienced dissociation as a seizure aura. Indeed, the authors found that this dissociation correlated with a 3 Hz rhythm in the deep posteromedial cortex. This area is the human analog to the mouse retrosplenial cortex. In addition, following electrical stimulation of the posteromedial cortex the patient consistently reported being in a dissociative state of mind. Merging their observations from the animal and the human experiments, the authors concluded this was evidence that a low-frequency rhythm in the deep posteromedial cortex is an evolutionarily conserved mechanism underlying dissociation across species.^100^ We can extrapolate that such mechanisms might be at play in humans with NDEs as well.

The evidence from all these candidate mechanisms has been combined into a ‘diathesis-stress model’.^11^^,^^14^^,^^101^ Thus, an unusually sensitive arousal system (i.e. the diathesis), as revealed by REM sleep intrusion, would predispose people to NDE in life-threatening situations and emotional stress.^11^^,^^101^ CSDs and NMDAR hypofunction could then be understood as contributing factors. This model seems consistent with the fact that the semiology of NDEs is identical in situations associated with real danger and the possibility for compromised brain physiology (e.g. cardiac arrest), situations associated with real danger but without impaired brain physiology (e.g. a near miss traffic accident), and situations where true danger is absent (e.g. meditation).^12^^,^^13^^,^^102–104^ Under any circumstances, people who are able to recall and report their NDEs many years later must have survived without any major brain damage. We suggest that the evolutionary aspects outlined in this paper can be added to this diathesis-stress model to account for the phylogenetic origin of NDEs.

### Limitations and strengths

Although we found evidence for thanatosis in all major taxa of our cladogram, we were unable to identify such reports in the great apes, i.e. gorillas, chimpanzees, bonobos and orangutans. However, dissociate states such as hypnosis do occur in e.g. chimpanzees,^105^ and since thanatosis is well-documented in macaques^51^^,^^52^ and humans,^25^^,^^70^ it seems unlikely that this trait would have been vanished in the great apes only to reoccur in humans. Of note, thanatosis in macaques has been described in captivity only. As apes are tree-dwellers, tonic immobility would be a disadvantage in most circumstances given the risk of falls. We therefore suggest this trait has been suppressed in tree-dwellers such as the great apes but not eradicated because it occurs in humans.

Furthermore, it should be noted that we used the terms ‘thanatosis’, ‘tonic immobility’ and ‘death-feigning’ interchangeably, which is commonly done in the scientific literature, but these terms contain a certain anthropomorphic bias. From an etymological point of view, for example, ‘to feign’ implies a conscious and deliberate act to deceive someone else, which seems an overinterpretation of insect behaviour.^72^ Similarly, ‘tonic’ immobility certainly happens (e.g. Fig. 1) but so does “atonic” immobility (e.g. Box 1, the grizzly bear narrative). As already mentioned, however, the common denominator for all this behaviour is the fact that the animal or human being under attack becomes immobile, which increases the chance of survival by preventing maladaptive behaviours such as panic or struggle which stimulate the predator.

Also, we were unable to identify survivors of big animal attacks with NDEs when contacting various organizations tracking such encounters like the Serengeti National Park. Our inquiries were usually either ignored or turned down without any explanation. In addition, our attempts to contact survivors via social media remained without results, and so did our attempts to identify reports of survivors with NDEs from the Holocaust and other genocides. Obviously, this does not prove that such reports do not exist, and a cursory online search revealed various reports of death feigning occurring in genocide survivors, as well as death feigning and NDEs in survivors of attacks by big animals. We hereby invite readers with NDEs who have survived such encounters to contact and tell us of their experience.

Finally, the Coma Science Group NDE database includes mostly reports from Western Europe and people who took the initiative to share their experience, so the number of 14% NDEs associated with ‘modern’ and human predators is biased. However, in an online survey of unprimed laypeople the rate of NDEs and NDE-like experiences associated with physical violence, excluding combat situations, was 8.3% (24/289 experiences); and the figures for combat situations and motor accidents were 3.8% (11/289) and 27% (77/289), respectively.^12^ These numbers are within the same order of magnitude as those from the Liège database. This suggests ‘modern’ and human predators are indeed a common cause for NDEs. That most NDEs occur in situations when no predator is involved is not surprising because other life-threatening events such as cardiac arrest or emergency surgery are much more frequent in humans.

As to the strengths of this paper, we used pre-specified work packages to investigate the association between thanatosis and NDEs from various perspectives, and we registered our protocol prior to data collection in order to avoid data cherry-picking.

## Conclusions and future directions

According to T. Dobzhansky (1900–75), ‘nothing in biology makes sense except in the light of evolution.’ To confirm that NDEs originate from thanatosis, prospective studies might inquire for tonic immobility in people taking the initiative to report their NDEs and unprimed laypeople. A more comprehensive search through the literature of the Holocaust and other genocides might uncover examples of NDEs, and NDEs from people with non-Western backgrounds need to be investigated for cultural variance. Furthermore, reports of thanatosis in great apes might be collected by contacting facilities where these animals are held in captivity, e.g. zoological gardens. Ultimately, the aim is to describe the genetic underpinnings of thanatosis and NDEs, which might be achievable by first focussing on taxa with relatively simple behaviours and genetic make-up like insects and then trying to identify risk loci in subsequently more complex animals, followed by humans. In summary, we have built a line of evidence suggesting thanatosis is the evolutionary foundation of NDEs. To our knowledge, no previous work has tried to provide such a phylogenetic basis. Hence, this may also be the first time we can assign a biological purpose to NDEs, which would be the benefit of survival.

## Abbreviations

CSD =: cortical spreading depolarization
NDE =: near-death experience
NMDAR =: N-methyl-d-aspartate receptor
REM =: rapid eye movement

## References

[1] Peinkhofer C , Dreier J , Kondziella D. Semiology and mechanisms of near-death experiences. Curr Neurol Neurosci Rep. 2019;19(9):62. 31352520 10.1007/s11910-019-0983-2

[2] Greyson B. Incidence and correlates of near-death experiences in a cardiac care unit. Gen Hosp Psychiatry. 2003;25(4):269–276. 12850659 10.1016/s0163-8343(03)00042-2

[3] Schwaninger J , Eisenberg PR , Schechtman KB , Weiss AN. A prospective analysis of near-death experiences in cardiac arrest patients. J Near Death Stud. 2002;20(4):215–232.

[4] van Lommel P , van Wees R , Meyers V , et al Near-death experience in survivors of cardiac arrest: A prospective study in the Netherlands. Lancet (London, England). 2001;358(9298):2039–2045. 10.1016/S0140-6736(01)07100-8 11755611

[5] Hou Y , Huang Q , Prakash R , Chaudhury S. Infrequent near death experiences in severe brain injury survivors - A quantitative and qualitative study. Ann Indian Acad Neurol. 2013;16(1):75–81. 23661968 10.4103/0972-2327.107715 PMC3644787

[6] Knoblauch H , Schmied I , Schnettler B. Todesnäheerfahrungen in Ost- und Westdeutschland : Eine empirische Untersuchung. In: Todesnähe: Wissenschaftliche Zugänge Zu Einem Außergewöhnlichen Phänomen. Konstanz, Germany: UVK;1999:217–250.

[7] Knoblauch H , Schmied I , Schnettler B. Different kinds of near-death experience: A report on a survey of near-death experiences in Germany. J Near Death Stud. 2001;20(1):15–29.

[8] Perera M , Padmasekara G , Belanti J. Prevalence of near-death experiences in Australia. J Near Death Stud. 2005;24(2):109–116.

[9] Timmermann C , Roseman L , Williams L , et al DMT models the near-death experience. Front Psychol. 2018;9:1424. 30174629 10.3389/fpsyg.2018.01424 PMC6107838

[10] Martial C , Cassol H , Charland-Verville V , et al Neurochemical models of near-death experiences: A large-scale study based on the semantic similarity of written reports. Conscious Cogn. 2019;69:52–69. 30711788 10.1016/j.concog.2019.01.011

[11] Nelson KR , Mattingly M , Lee SA , Schmitt FA. Does the arousal system contribute to near death experience? Neurology. 2006;66(7):1003–1009. 16606911 10.1212/01.wnl.0000204296.15607.37

[12] Kondziella D , Dreier JP , Olsen MH. Prevalence of near-death experiences in people with and without REM sleep intrusion. PeerJ. 2019;7:e7585. 31523519 10.7717/peerj.7585 PMC6716500

[13] Kondziella D , Olsen MH , Lemale CL , Dreier JP. Migraine aura, a predictor of near-death experiences in a crowdsourced study. PeerJ. 2019;7:e8202. 31824781 10.7717/peerj.8202 PMC6898989

[14] Kondziella D. The neurology of death and the dying brain: A pictorial essay. Front Neurol. 2020;11:736. 32793105 10.3389/fneur.2020.00736 PMC7385288

[15] Lake J. The near-death experience (NDE) as an inherited predisposition: Possible genetic, epigenetic, neural and symbolic mechanisms. Med Hypotheses. 2019;126:135–148. 31010490 10.1016/j.mehy.2019.03.016

[16] Evrard R , Toutain C , Glazier JW , Le Maléfan P. The energy of despair: Do near-death experiences have an evolutionary value? Psychol Conscious Theory Res Pract. 2018;6:184–199.

[17] Gabrielsen GW , Smith EN. Physiological responses associated with feigned death in the American opossum. Acta Physiol Scand. 1985;123(4):393–398. 3993399 10.1111/j.1748-1716.1985.tb07605.x

[18] Humphreys RK , Ruxton GD. A review of thanatosis (death feigning) as an anti-predator behaviour. Behav Ecol Sociobiol. 2018;72(2):22. 29386702 10.1007/s00265-017-2436-8 PMC5769822

[19] Kiyotake H , Matsumoto H , Nakayama S , et al Gain of long tonic immobility behavioral trait causes the red flour beetle to reduce anti-stress capacity. J Insect Physiol. 2014;60:92–97. 24291367 10.1016/j.jinsphys.2013.11.008

[20] van de Kamp T , Cecilia A , dos Santos Rolo T , Vagovič P , Baumbach T , Riedel A. Comparative thorax morphology of death-feigning flightless cryptorhynchine weevils (Coleoptera: Curculionidae) based on 3D reconstructions. Arthropod Struct Dev. 2015;44(6 Pt A):509–523. 26259678 10.1016/j.asd.2015.07.004

[21] Gregory PT , Isaac LA , Griffiths RA. Death feigning by grass snakes (*Natrix natrix*) in response to handling by human “predators.” J Comp Psychol. 2007;121(2):123–129. 17516791 10.1037/0735-7036.121.2.123

[22] Zamudio SR , Quevedo-Corona L , Garcés L , De La Cruz F. The effects of acute stress and acute corticosterone administration on the immobility response in rats. Brain Res Bull. 2009;80(6):331–336. 19772903 10.1016/j.brainresbull.2009.09.005

[23] Donatti AF , Leite-Panissi CRA , Ferreira A , Ramos C , Leite P. A. Activation of corticotropin-releasing factor receptors from the basolateral or central amygdala increases the tonic immobility response in guinea pigs: An innate fear behavior. Behav Brain Res. 2011;225(1):23–30. 21741994 10.1016/j.bbr.2011.06.027

[24] Kimble DP. Didelphid behavior. Neurosci Biobehav Rev. 1997;21(3):361–369. 9168270 10.1016/s0149-7634(96)00016-4

[25] Kalaf J , Vilete LMP , Volchan E , et al Peritraumatic tonic immobility in a large representative sample of the general population: Association with posttraumatic stress disorder and female gender. Compr Psychiatry. 2015;60:68–72. 25891640 10.1016/j.comppsych.2015.04.001

[26] TeBockhorst SF , O'Halloran MS , Nyline BN. Tonic immobility among survivors of sexual assault. Psychol Trauma. 2015;7(2):171–178. 25793694 10.1037/a0037953

[27] Kozlowska K , Walker P , McLean L , Carrive P. Fear and the defense cascade: Clinical implications and management. Harv Rev Psychiatry. 2015;23(4):263–287. 26062169 10.1097/HRP.0000000000000065 PMC4495877

[28] Bastos AF , Vieira AS , Oliveira JM , et al Stop or move: Defensive strategies in humans. Behav Brain Res. 2016;302:252–262. 26802729 10.1016/j.bbr.2016.01.043

[29] Skelhorn J. Avoiding death by feigning death. Curr Biol. 2018;28(19):R1135–R1136. 30300594 10.1016/j.cub.2018.07.070

[30] Rogers SM , Simpson SJ. Thanatosis. Curr Biol. 2014;24(21):R1031–R1033. 25517363 10.1016/j.cub.2014.08.051

[31] Cook DR , Smith AT , Proud DN , Víquez C , Townsend VR. Defensive responses of neotropical harvestmen (Arachnida, Opiliones) to generalist invertebrate predators. Caribb J Sci. 2013;47(2-3):325–334.

[32] Jones TC , Akoury TS , Hauser CK , et al Octopamine and serotonin have opposite effects on antipredator behavior in the orb-weaving spider, *Larinioides cornutus*. J Comp Physiol A Neuroethol Sens Neural Behav Physiol. 2011;197(8):819–825. 21484264 10.1007/s00359-011-0644-7

[33] Miyatake T , Katayama K , Takeda Y , Nakashima A , Sugita A , Mizumoto M. Is death-feigning adaptive? Heritable variation in fitness difference of death-feigning behaviour. Proc R Soc B Biol Sci. 2004;271(1554):2293–2296. 10.1098/rspb.2004.2858 PMC1691851 15539355

[34] O'Brien TJ , Dunlap WP. Tonic immobility in the blue crab (Callinectes sapidus, Rathbun) its relation to threat of predation. J Comp Physiol Psychol. 1975;89(1):86–94. 1171124 10.1037/h0076425

[35] Cazzolla Gatti R , Messina G , Tiralongo F , Ursino LA , Lombardo BM. Learning from the environment: How predation changes the behavior of terrestrial Isopoda (Crustacea Oniscidea). Ethol Ecol Evol. 2020;32(1):29–45.

[36] Henningsen AD. Tonic immobility in 12 elasmobranchs: Use as an aid in captive husbandry. Zoo Biol. 1994;13(4):325–332.

[37] Williamson MJ , Dudgeon C , Slade R. Tonic immobility in the zebra shark, *Stegostoma fasciatum*, and its use for capture methodology. Environ Biol Fishes. 2018;101(5):741–748.

[38] Lefebvre L , Sabourin M. Effects of spaced and massed repeated elicitation on tonic immobility in the goldfish (*Carassius auratus*). Behav Biol. 1977;21(2):300–305.

[39] Freret-Meurer NV , Fernandez TC , Lopes DA , Vaccani AC , Okada NB. Thanatosis in the Brazilian seahorse *Hippocampus reidi* Ginsburg, 1933 (Teleostei: Syngnathidae). Acta Ethol. 2017;20(1):81–84.

[40] Toledo LF , Sazima I , Haddad CFB. Behavioural defences of anurans: An overview. Ethol Ecol Evol. 2011;23(1):1–25.

[41] Passos LF , Garcia G , Young RJ. The tonic immobility test: Do wild and captive golden mantella frogs (*Mantella aurantiaca*) have the same response? PLoS One. 2017;12(7):e0181972. 28732029 10.1371/journal.pone.0181972 PMC5521826

[42] Mesquita GDS , Ferraz D , Ramalho WP. Death-feigning as defensive behavior in blue-tailed microteiid lizard *Micrablepharus atticolus* Rodrigues, 1996. Herpetol Notes. 2018;11:1065–1067.

[43] Francq EN. Behavioral aspects of feigned death in the opossum *Didelphis marsupialis*. Am Midl Nat. 1969;81(2):556–568.

[44] Moore AU , Amstey MS. Tonic immobility: Differences in susceptibility of experimental and normal sheep and goats. Science (80-). 1962;135(3505):729–730. 10.1126/science.135.3505.729 14475631

[45] Lundgren EJ , Moeller KT , Pecari P. Anti-predator strategies of, and possible thanatosis in, juvenile collared peccaries (*Pecari tajacu*). Southwest Nat. 2017;62(3):235–237.

[46] Hudson WH. The Naturalist In La Plata. New York, NY, US: D. Appleton and Company; 1985.

[47] Reese WG , Newton JEO , Angel C. Immobility experiments with dogs of the Arkansas Line of Nervous Pointers. Pavlov J Biol Sci off J Pavlov. 1985;20(3):132–139. 10.1007/BF03003596 4041028

[48] Webster DG , Lanthorn TH , Dewsbury DA , Meyer ME. Tonic immobility and the dorsal immobility response in twelve species of muroid rodents. Behav Neural Biol. 1981;31(1):32–41. 7305807 10.1016/s0163-1047(81)91034-7

[49] Ewell AH Jr , Cullen JM , Woodruff ML. Tonic immobility as a predator-defense in the rabbit (*Oryctolagus cuniculus*). Behav Neural Biol. 1981;31(4):483–489.

[50] Giannico AT , Lima L , Lange RR , et al Proven cardiac changes during death-feigning (tonic immobility) in rabbits (*Oryctolagus cuniculus*). J Comp Physiol A Neuroethol Sens Neural Behav Physiol. 2014;200(4):305–310. 24515628 10.1007/s00359-014-0884-4

[51] Foley JP. Tonic immobility in the rhesus monkey (Macaca mulatta) induced by manipulation, immobilization, and experimental inversion of the visual field. J Comp Psychol. 1938;26(3):515–526.

[52] Holcombe V , Sterman MB , Goodman SJ , Fairchild MB. The immobilization response in rhesus monkey: A behavioral and electroencephalographic study. Exp Neurol. 1979;63(2):420–435. 108127 10.1016/0014-4886(79)90136-5

[53] Thompson RKR , Foltin RW , Boylan RJ , Sweet A , Graves CA , Lowitz CE. Tonic immobility in Japanese quail can reduce the probability of sustained attack by cats. Anim Learn Behav. 1981;9(1):145–149.

[54] Sargeant AB , Eberhardt LE. Death feigning by ducks in response to predation by red foxes (*Vulpes fulva*). Am Midl Nat. 1975;94(1):108–119.

[55] Herzog HA. Immobility in intraspecific encounters: Cockfights and the evolution of “Animal hypnosis”. Psychol Rec. 1978;28(4):543–548.

[56] Hennig CW. Tonic immobility in the squirrel monkey (*Saimiri sciureus*). Primates. 1978;19(2):333–342.

[57] Berkhoff KC. Dina Pronicheva’s story of surviving the Babi Yar Massacre: German, Jewish, Soviet, Russian and Ukrainian Records. In: Brandon R , Lower W , eds. The Shoah in Ukraine: History, testimony, and memorialization. Bloomington, Indiana, USA: Indians University Press; 2010.

[58] Braham RL. The Szalazi Era. In: The Politics of Genocide. The Holocaust in Hungary. New York, NY, USA: Columbia University Press; 1981:872.

[59] Khamis J. Rwanda genocide 25 years on: “I pretended I was dead, I lay there all night, all I can remember is the moonlight and smell of blood.” Gulfnews. 2019; https://gulfnews.com/world/africa/rwanda-genocide-25-years-on-i-pretended-i-was-dead-i-lay-there-all-night-all-i-can-remember-is-the-moonlight-and-smell-of-blood-1.1554650281911. Accessed 21 June 2021.

[60] Avdic N. Survivor Recalls Srebrenica Horror. Radio Free Europe Radio Liberty. 2017. https://www.rferl.org/a/bosnia-srebrenica-survivor/28606557.html. Accessed 21 June 2021.

[61] Anonymous ANF. Twenty years on, Srebrenica survivor remembers hours in hell. AhramOnline. 2015. https://www.justiceinfo.net/en/1123-twenty-years-on-srebrenica-survivor-remembers-hours-in-hell.html.

[62] Cerkez A. Survivor recounts horror of Bosnia’s killing fields. New York, NY, USA: Assoc Press; 2011. https://www.nbcnews.com/id/wbna43272062. Accessed 21 June 2021.

[63] Townsend M , McVeigh T. Utøya, the island paradise turned into hell by Anders Behring Breivik. Norway: The Guardian: The Observer; 2011.

[64] Filkuková P , Hafstad GS , Jensen TK. Who can I trust? Extended fear during and after the Utøya terrorist attack. Psychol Trauma Theory Res Pract Policy. 2016;8(4):512–519. 10.1037/tra0000141 27124617

[65] Healy J , Santora M. Held Hostage in an Orlando Restroom, and Playing Dead to Stay Alive. *The New York Times*. 2016:1.

[66] Bilska A , Francikowski J , Wyglenda A , Masłowski A , Kaszyca N , Depa Ł. Aphids playing possum - Defensive or mutualistic response? J Insect Behav. 2018;31(1):42–53. 29527095 10.1007/s10905-018-9662-4 PMC5834575

[67] Neves FM , Pie MR. On the adult behavioral repertoire of the sawfly *Perreyia flavipes* Konow, 1899 (Hymenoptera: Pergidae): Movement, mating, and thanatosis. Neotrop Entomol. 2018;47(1):46–52. 28326460 10.1007/s13744-017-0509-z

[68] Segovia JMG , Murayama GP , Willemart RH. Sexual differences in weaponry and defensive behavior in a neotropical harvestman. Curr Zool. 2019;65(5):553–558. 31616486 10.1093/cz/zoy073 PMC6784509

[69] Bilde T , Tuni C , Elsayed R , Pekár S , Toft S. Death feigning in the face of sexual cannibalism. Biol Lett. 2006;2(1):23–25. 17148316 10.1098/rsbl.2005.0392 PMC1617195

[70] Marx BP , Forsyth JP , Gallup GG , Fusé T , Lexington JM. Tonic immobility as an evolved predator defense: Implications for sexual assault survivors. Clin Psychol Sci Pract. 2008;15(1):74–90.

[71] Bracha HS. Freeze, flight, fight, fright, faint: Adaptationist perspectives on the acute stress response spectrum. CNS Spectr. 2004;9(9):679–685. 15337864 10.1017/s1092852900001954

[72] Konishi K , Matsumura K , Sakuno W , Miyatake T. Death feigning as an adaptive anti‐predator behavior: Further evidence for its evolution from artificial selection and natural populations. J Evol Biol. 2020;33(8):1120–1128. 10.1111/jeb.13641 32426887

[73] Darwin CR. Essay on Instinct. In: Romanes GJ , ed. Mental evolution in animals. With a posthumous essay on instinct by Charles Darwin. London, UK: Kegan Paul Trenc & Co; 1883:335–384.

[74] Hay A. Surviving the impossible: The long march from Srebrenica. An investigation of the possible use of chemical warfare agents. Med Confl Surviv. 1998;14(2):120–155. doi:10.1080/13623699808409383 9633268 10.1080/13623699808409383

[75] Pfister O. Schockdenken und Schockphantasien bei höchster Lebensgefahr. Int Zeitschrift für Psychoanal. 1930;16:340–455.

[76] Noyes R. The encounter with life-threatening danger: Its nature and essence. Essence (Downsview). 1981;5:21–32.

[77] Krishnan V. Near-death experiences: Reassessment urged. Parapsychol Rev. 1981;12:10–11.

[78] Greyson B. The psychodynamics of near-death experiences. J Nerv Ment Dis. 1983;171(6):376–381.doi:10.1097/00005053-198306000-00008 6343557 10.1097/00005053-198306000-00008

[79] Ishijima K , Negayama K. Development of mother–infant interaction in tickling play: The relationship between infants’ ticklishness and social behaviors. Infant Behav Dev. 2017;49:161–167. 28934614 10.1016/j.infbeh.2017.08.007

[80] Ishiyama S , Kaufmann LV , Brecht M. Behavioral and cortical correlates of self-suppression, anticipation, and ambivalence in rat tickling. Curr Biol. 2019;29(19):3153–3164.e3. 31564493 10.1016/j.cub.2019.07.085

[81] van Berlo E , Díaz-Loyo AP , Juárez-Mora OE , Kret ME , Massen JJM. Experimental evidence for yawn contagion in orangutans (*Pongo pygmaeus*). Sci Rep. 2020;10(1):22251 33335177 10.1038/s41598-020-79160-x PMC7747555

[82] Dreier JP. The role of spreading depression, spreading depolarization and spreading ischemia in neurological disease. Nat Med. 2011;17(4):439–447. 21475241 10.1038/nm.2333

[83] Dreier JP , Major S , Foreman B , et al Terminal spreading depolarization and electrical silence in death of human cerebral cortex. Ann Neurol. 2018;83(2):295–310. 29331091 10.1002/ana.25147 PMC5901399

[84] Carlson AP , Shuttleworth CW , Major S , Lemale CL , Dreier JP , Hartings JA. Terminal spreading depolarizations causing electrocortical silencing prior to clinical brain death: Case report. J Neurosurg. 2018;131(6):1–7. 10.3171/2018.7.JNS181478 30544340

[85] Dreier JP , Kleeberg J , Petzold G , et al Endothelin-1 potently induces Leão’s cortical spreading depression in vivo in the rat: A model for an endothelial trigger of migrainous aura? Brain. 2002;125(Pt 1):102–112. 11834596 10.1093/brain/awf007

[86] Spong KE , Dreier JP , Robertson RM. A new direction for spreading depolarization: Investigation in the fly brain. Channels (Austin). 2017;11(2):97–98. 27657932 10.1080/19336950.2016.1239898 PMC5398573

[87] Farkas E , Pratt R , Sengpiel F , Obrenovitch TP. Direct, live imaging of cortical spreading depression and anoxic depolarisation using a fluorescent, voltage-sensitive dye. J Cereb Blood Flow Metab off J Int Soc Cereb Blood Flow Metab. 2008;28(2):251–262. 10.1038/sj.jcbfm.9600569 PMC2653938 17971792

[88] Saper CB. The neurobiology of sleep. Contin Lifelong Learn Neurol. 2013;19(1 Sleep Disorders):19–31. 10.1212/01.CON.0000427215.07715.73 23385692

[89] Grace KP , Horner RL. A focal inactivation and computational study of ventrolateral periaqueductal gray and deep mesencephalic reticular nucleus involvement in sleep state switching and bistability. eNeuro. 2020;7(6):ENEURO.0451-19.2020. 10.1523/ENEURO.0451-19.2020 PMC7768273 33055199

[90] Lu J , Sherman D , Devor M , Saper CB. A putative flip-flop switch for control of REM sleep. Nature. 2006;441(7093):589–594. 16688184 10.1038/nature04767

[91] Bandler R , Keay KA , Floyd N , Price J. Central circuits mediating patterned autonomic activity during active vs. passive emotional coping. Brain Res Bull. 2000;53(1):95–104. 11033213 10.1016/s0361-9230(00)00313-0

[92] Keay KA , Bandler R. Parallel circuits mediating distinct emotional coping reactions to different types of stress. Neurosci Biobehav Rev. 2001;25(7-8):669–678. 11801292 10.1016/s0149-7634(01)00049-5

[93] Scammell TE. Narcolepsy. In Campion EW , ed. N Engl J Med. 2015;373(27):2654–2662. 26716917 10.1056/NEJMra1500587

[94] Scammell TE , Arrigoni E , Lipton JO. Neural circuitry of wakefulness and sleep. Neuron. 2017;93(4):747–765. 28231463 10.1016/j.neuron.2017.01.014 PMC5325713

[95] Leung LC , Wang GX , Madelaine R , et al Neural signatures of sleep in zebrafish. Nature. 2019;571(7764):198–204. 31292557 10.1038/s41586-019-1336-7 PMC7081717

[96] Chindo BA , Adzu B , Yahaya TA , Gamaniel KS. Ketamine-enhanced immobility in forced swim test: A possible animal model for the negative symptoms of schizophrenia. Prog Neuropsychopharmacol Biol Psychiatry. 2012;38(2):310–316. 22561603 10.1016/j.pnpbp.2012.04.018

[97] Rogers JP , Pollak TA , Blackman G , David AS. Catatonia and the immune system: A review. Lancet Psychiatry. 2019;6(7):620–630. 31196793 10.1016/S2215-0366(19)30190-7 PMC7185541

[98] Krystal JH , Karper LP , Seibyl JP , et al Subanesthetic effects of the noncompetitive NMDA antagonist, ketamine, in humans. Psychotomimetic, perceptual, cognitive, and neuroendocrine responses. Arch Gen Psychiatry. 1994;51(3):199–214. 8122957 10.1001/archpsyc.1994.03950030035004

[99] Al-Diwani A , Handel A , Townsend L , et al The psychopathology of NMDAR-antibody encephalitis in adults: A systematic review and phenotypic analysis of individual patient data. Lancet Psychiatry. 2019;6(3):235–246. 30765329 10.1016/S2215-0366(19)30001-X PMC6384244

[100] Vesuna S , Kauvar IV , Richman E , et al Deep posteromedial cortical rhythm in dissociation. Nature. 2020;586(7827):87–94. 32939091 10.1038/s41586-020-2731-9 PMC7553818

[101] Long J , Holden JM. Does the arousal system contribute to near-death and out-of body experiences? A summary and response. J Near Death Stud. 2007;25(3):135–169.

[102] Cassol H , Martial C , Annen J , et al A systematic analysis of distressing near-death experience accounts. Memory. 2019;27(8):1122–1128. 31189413 10.1080/09658211.2019.1626438

[103] Martial C , Charland-Verville V , Dehon H , Laureys S. False memory susceptibility in coma survivors with and without a near-death experience. Psychol Res. 2018;82(4):806–818. 28303355 10.1007/s00426-017-0855-9

[104] Martial C , Cassol H , Antonopoulos G , et al Temporality of features in near-death experience narratives. Front Hum Neurosci. 2017;11:311. 28659779 10.3389/fnhum.2017.00311 PMC5469194

[105] Völgyesi FA. Hypnosis of man and animals: With special rederence to development of the brain in the species and in the individual, 2nd ed. London, UK: Bailliére, Tindall & Cassell; 1966.

[106] Grose J. I survived a bear attack. *Slate*. 2012. https://slate.com/technology/2012/04/grizzly-attack-victim-interview-with-survivor-deb-freele.html. Accessed 21 June 2021.

[107] Livingstone D. Missionary travels and researches in South Africa. London, UK: John Murray; 1857.

[108] Steiger B , Steiger SH. Real encounters, different dimensions and otherworldly beigns. Canton, Michigan, USA: Visible Ink Press; 2013.

[109] Nguyen CT. In interview, Horn describes near-death experience. LasVegasSun. 2004. https://lasvegassun.com/news/2004/sep/16/in-interview-horn-describes-near-death-experience/. Accessed 21 June 2021.

[110] Nderf.org. https://www.nderf.org/Experiences/1neha_s_nde.html. NDERF stories website.

[111] https://www.youtube.com/watch?v=JqlGjX1MtVg. ContentMint. 2011. Gazelle’s LUCKY ESCAPE from CHEETAH and HYENA by PLAYING DEAD! [Video]. Youtube. https://youtu.be/Lupt2qajcJg. Accessed 9 August 2011.

[112] www.youtube.com/watch?v=pEVPBO-XF0o&t=40s. Kruger Sightings. *Hyena* Indirectly Saves Impala from Leopard. [Video]. 2018. https://youtu.be/pEVPBO-XF0o. Accessed 27 February 2021.

[113] www.youtube.com/watch?v=Ox7Uj2pw-80. Jim Hopper.Impala in and slowly out of collapsed immobility.[Video]. 2017. https://youtu.be/Ox7Uj2pw-80. Accessed 27 February 2021.

[114] www.youtube.com/watch?v=K-DWmNnnWbE. Kruger Sightings. *Wild* Dog Plays Dead to Escape Lion. [Video]. 2019. https://youtu.be/K-DWmNnnWbE. Accessed 27 February 2021..

